# Avoiding tension in left internal mammary artery to left anterior descending coronary artery anastomosis during coronary artery bypass graft surgery

**DOI:** 10.1308/003588413X13511609957056

**Published:** 2013-01

**Authors:** KMJ Chan, OA Jarral, RA Jarral, PP Punjabi

**Affiliations:** Imperial College Healthcare NHS Trust, UK

*The authors have justified to the Editor the contribution made by each author. All authors reviewed and approved the final version*.

## Background

The left internal mammary artery (LIMA) to left anterior descending coronary artery (LAD) graft is the most important graft in coronary artery bypass graft surgery.^1^ The length of the LIMA is fixed and situations may arise when this is inadequate.^2^ This may not be apparent during the cardiopulmonary bypass until after the anastomosis has been performed and the lungs re-expanded. We describe a technique to reduce the tension on the LIMA to LAD anastomosis in such situations.

## Technique

**Figure 1:**
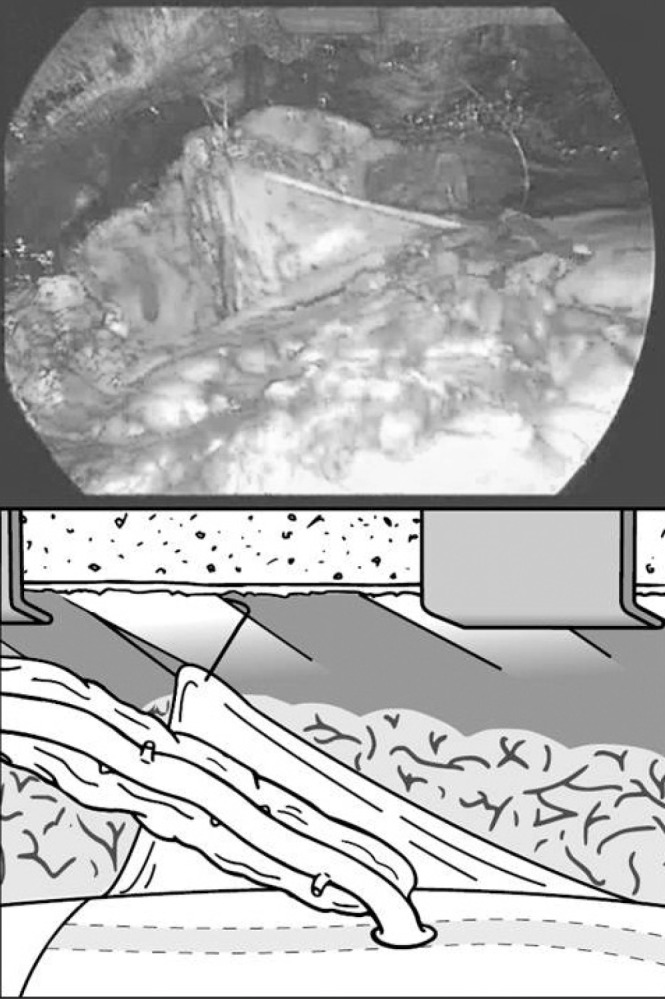
Top: operative photograph taken from the right of the chest showing the left internal mammary artery (LIMA) lying on the anterior surface of the heart anastomosed to the left anterior descending coronary artery (LAD). The inferior limb of the left pericardium is shown stitched to the costal cartilage, lifting the heart anteriorly and caudally and preventing hyperexpansion of the lung from causing tension on the LIMA to LAD anastomosis. Bottom: a schematic representation of this

A vertical slit is made in the pericardium, creating a short superior limb and an inferior limb continuous with the rest of the pericardium. The inferior limb of the pericardial slit is lifted caudally and laterally and sutured to a costal cartilage as caudally as possible (approximately two intercostal spaces caudal to its original position and just medial to the lateral margin of the LIMA harvest site) using a 2/0 Ethibond^®^ suture (Ethicon, Somerville, NJ, US) (Fig 1). This manoeuvre pulls the heart anteriorly towards the chest wall and caudally, thereby reducing the tension on the LIMA to LAD anastomosis. It also prevents the hyperinflated lungs stretching the LIMA and exerting tension on it.

## Discussion

We have used this technique successfully in a few cases where we have found the LIMA to be under likely tension or were concerned with emphysematous lungs stretching the LIMA. This simple and easily reproducible technique, which has also been used by others, is a helpful solution to an occasional problem.
